# Auditory biological marker of concussion in children

**DOI:** 10.1038/srep39009

**Published:** 2016-12-22

**Authors:** Nina Kraus, Elaine C. Thompson, Jennifer Krizman, Katherine Cook, Travis White-Schwoch, Cynthia R. LaBella

**Affiliations:** 1 Auditory Neuroscience Laboratory, Northwestern University, Evanston, IL, United States.; 2 Department of Communication Sciences, Northwestern University, Evanston, IL, United States.; 3 Department of Neurobiology & Physiology, Northwestern University, Evanston, IL, United States.; 4 Department of Otolaryngology, Northwestern University’s Feinberg School of Medicine, Chicago, IL, United States.; 5 Division of Pediatric Orthopaedic Surgery & Sports Medicine, Ann & Robert H. Lurie Children’s Hospital of Chicago, Chicago, IL, United States.; 6 Department of Pediatrics, Northwestern University’s Feinberg School of Medicine, Chicago, IL, United States

## Abstract

Concussions carry devastating potential for cognitive, neurologic, and socio-emotional disease, but no objective test reliably identifies a concussion and its severity. A variety of neurological insults compromise sound processing, particularly in complex listening environments that place high demands on brain processing. The frequency-following response captures the high computational demands of sound processing with extreme granularity and reliably reveals individual differences. We hypothesize that concussions disrupt these auditory processes, and that the frequency-following response indicates concussion occurrence and severity. Specifically, we hypothesize that concussions disrupt the processing of the fundamental frequency, a key acoustic cue for identifying and tracking sounds and talkers, and, consequently, understanding speech in noise. Here we show that children who sustained a concussion exhibit a signature neural profile. They have worse representation of the fundamental frequency, and smaller and more sluggish neural responses. Neurophysiological responses to the fundamental frequency partially recover to control levels as concussion symptoms abate, suggesting a gain in biological processing following partial recovery. Neural processing of sound correctly identifies 90% of concussion cases and clears 95% of control cases, suggesting this approach has practical potential as a scalable biological marker for sports-related concussion and other types of mild traumatic brain injuries.

Concussions are a public health crisis. An estimated 1.6–3.8 million sports-related traumatic brain injuries occur annually in the United States^1^, and concussions—diffuse, non-penetrating brain injuries following sudden impact—potentially devastate cognition, socioemotional wellbeing, academic achievement, and neurologic function, even after symptoms resolve^2^,^3^. Despite widespread scientific and public interest, no single test has been validated to reliably diagnose a concussion; instead, the gold standard for diagnosis remains clinical determination by a physician who must weigh a constellation of symptoms across multiple organ systems.

The answer may be in the auditory system. Among the consequences of mild traumatic brain injuries (mTBI) such as concussions is a compromised ability to make sense of sound. This challenge is manifest in the complex listening environments that pervade everyday life, where difficulty making meaning from sound curtails opportunities for learning and social bonding^4^. Active listening engages a diverse series of brain networks; insults to any component may compromise listening, particularly the ability to understand complex signals such as speech. Support for this idea comes from studies of aging^5^, language impairment^6^, HIV-associated neurocognitive disorders^7^, and, indeed, mTBI^8^,^9^.

How might concussions disrupt auditory processing? The fundamental frequency (F_0_) of sound is a chief acoustic cue for everyday listening; tracking the F_0_ facilitates pitch perception, identifying sounds and talkers, and understanding stress and prosody. It should come as no surprise, then, that individual differences in the neurophysiological processing of the F_0_ cascade to individual differences in listening skills: across the lifespan, listeners with stronger responses to the F_0_ hear better in everyday environments^10^,^11^,^12^. Thus, we spotlight the F_0_ in the biological approach considered here.

Our conceptual framework positions auditory processing—the ability to automatically and efficiently extract meaning from sound—at the nexus of cognitive, sensory, and limbic systems, and argues that insults to any of these domains undermine sound processing^13^. Sound processing is one of the most computationally-demanding tasks the nervous system has to perform, relying on the exquisite timing found in the auditory system, which responds to input more than one thousand times more quickly than photoreceptors in the visual system. Thus, the auditory system may be sensitive to neurological insults that disrupt microsecond-level temporal resolution.

The current standard for concussion diagnosis is largely subjective in that it relies on accurate symptom reporting by the patient. Thus, there are ongoing efforts to identify objective markers to assist in diagnosing a concussion and predicting recovery. One area of focus is on cerebrospinal fluid- and blood-based biomarkers that test for sequelae of neural injury. For example, Olivera *et al*.^14^ reported that plasma tau protein levels were elevated in military personnel with self-reported TBI, with the highest levels in individuals with multiple TBIs. However, these biomarkers are invasive and may not extend to milder forms of TBI, such as concussions. A second area tries to adopt neuroimaging techniques, such as diffusion tensor imaging and functional magnetic resonance imaging, to detect concussions. However, these approaches rely on expensive equipment and contradictory results are often reported: for example, both increases and decreases in white matter volume have been associated with mTBI^15^.

Visual, auditory, and somatosensory evoked potentials have all been explored in individuals following head injury, but these techniques seem to reflect *severe* TBI and coma as opposed to mild TBI such as concussions (for review see ref. 16). Promising, but contradictory, findings have been reported for auditory-evoked potentials^16^,^17^,^18^. Also challenging is the wide within and between subject variability seen in these evoked potentials, making them unreliable clinical tools; moreover, some measures can be unpredictably affected by sleep and subject awareness^19^. Overall, current neuroimaging and electrophysiological approaches for concussion show *group* differences but overlap between groups potentially thwarts evaluation of *individual* differences. Heterogeneity of approaches, clinical criteria, inclusion/exclusion criteria, and severity of the TBI also means that these studies do not replicate robustly.

The limitations of the aforementioned approaches necessitate a fresh methodology that has granularity into the biological minutiae of sound processing, and one that reliably indicates individual differences. Because we ultimately want an approach that may be clinically adoptable, we want a test that is fast, mobile, objective, and applicable to *mild* TBI. Thus, we measured speech-evoked frequency-following responses (FFRs; also called the auditory brainstem response to complex sounds, or cABR^20^) in children diagnosed with a concussion.

The FFR achieves these goals because it reflects the cognitive, sensorimotor, and reward networks fundamental to everyday listening^13^, and has potential to predict outcomes in clinical populations^21^. The FFR is generated predominantly by the auditory midbrain, a site of converging ascending and descending inputs across multiple neural systems; thus, the FFR could reflect neurological insults originating beyond the auditory system, including those endemic to a concussion. Noteworthy is that concussions and their sequelae are associated with demyelination, axonal injury, and excessive extracellular tau protein in subcortical systems^22^,^23^, in addition to temporal and frontal cortices (thought to innervate the auditory midbrain directly^24^). FFR enables examination of amplitude, timing, frequency representation, and accuracy within a single fast-to-administer assay. From a clinical perspective, it is also important to point out that the FFR is highly reliable (test-retest reliability often exceeding *r* = 0.9), may be applied in a uniform manner across the lifespan, and has established norms that show sub-millisecond timing differences are meaningful^25^. The purpose of our study was to determine whether FFRs are a valid and reliable biomarker for concussion in children.

Here, we recruited children from a sports medicine clinic, each of whom had been diagnosed with a concussion, and compared them to a matched control group. We also followed a subset of children in the concussion group longitudinally. We tested four specific predictions. First, we predicted that auditory processing, as measured by the FFRs, should be distinct in children who have sustained a concussion when compared to age- and sex-matched controls. We specifically predicted that the children with a concussion would have poorer neural processing of the fundamental frequency of speech (F_0_). Second, we predicted that the symptom load of a concussion would track the integrity of biological sound processing, as measured by the FFR. Third, we predicted that FFRs could be harnessed as a biological marker to identify which children in our cohort sustained a concussion. Finally, we predicted that auditory processing should improve as children recover from their injuries.

We measured FFRs to the speech sound /d/ in groups of children with and without a concussion (Fig. 1A). Complex sounds such as speech contain an F_0_ (that typically conveys the perception of pitch) that facilitates sound identification/auditory object formation and grouping, and *harmonics* (integer harmonic frequencies of the F_0_) that, in speech, contribute phonemic information. Models of speech production and perception distinguish these features^26^,^27^,^28^,^29^; given our hypothesis that F_0_ processing is disrupted, our analyses focused on measures of how accurately, quickly, and robustly the F_0_ is processed. We compared these to measures of harmonic processing, to ascertain if concussions impart a global decline in the FFR or if it is indeed specific to the F_0_.

## Results

### Neural coding of the fundamental frequency is disrupted following concussion

**Figure 1:**
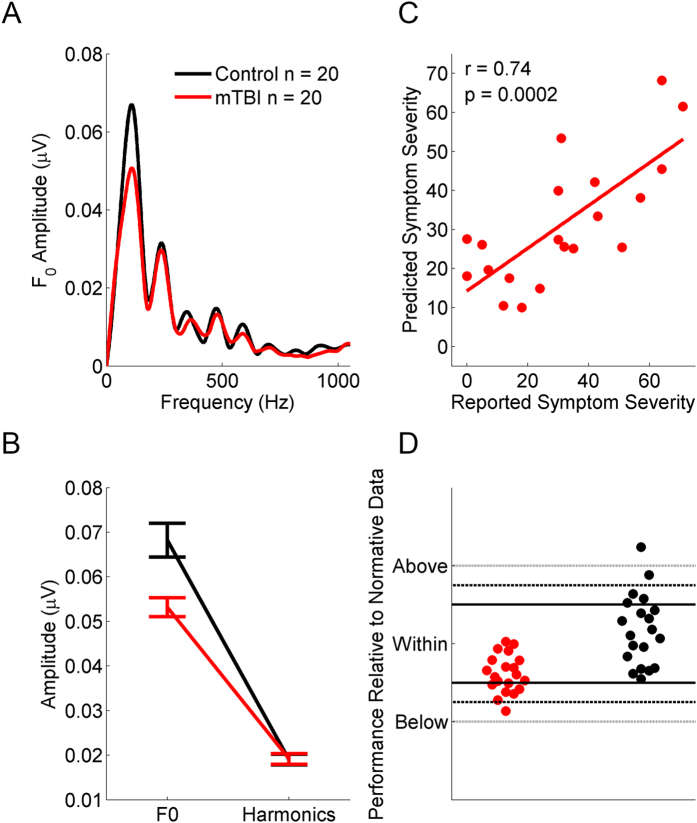
The neural coding of the fundamental frequency (F_0_, peak at around 100 Hz), but not harmonic (peaks from 200 to 1000 Hz), cues is impaired in children with a concussion. (**A**,**B**) The concussed children (red) have smaller responses to the pitch of a talker’s voice than their non-concussed peers (black). A regression predicting symptom load from neural processing of the F_0_ (controlling for sex) illustrates a high degree of similarity between reported and predicted symptoms (**C**) and the majority of children in the concussion group are at or below the 50^th^ percentile (**D**) relative to established norms^25^ (B: Error bars represent ± 1 S.E.M.; D: Horizontal solid lines represent ±1 SD of normative data, horizontal dashed lines represent ± 1.5 SDs of normative data, and horizontal dotted lines represent 2 SDs of normative data).

The children who sustained a concussion had smaller responses to the F_0_ than their peers in the control group (by ≈ 35%), but the groups had similar harmonic processing (group × frequency interaction, *F*(1, 38) = 16.554, p < 0.001, η^2^ = 0.303; *post-hoc* group differences for F_0_, *t*(38) = 3.607, *p* = 0.001, Cohen’s *d* = 1.223; harmonics, *t*(38) = 1.056, *p* = 0.298, Cohen’s *d* = 0.329; Fig. 1A/B). To complement this analysis, we determined the strength of pitch coding by performing autocorrelations on the FFRs^30^. Children with a concussion had poorer pitch coding than their peers from the control group (*t*(38) = 2.773, *p* = 0.009, Cohen’s *d* = 1.14; see Table 1).

Within the concussion group, children who reported the highest symptom load had the smallest responses to the F_0_ (regression controlling for sex, *R*^2^ = 0.548, *F*(2, 19) = 10.287, *p* = 0.001; *β*_F0_ = −0.712, *p* = 0.001).

### Diminished neural responses to speech following concussion

**Figure 2:**
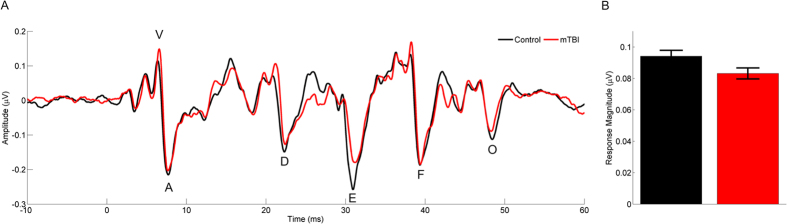
Children with a concussion have smaller and slower neural responses to speech. Comparison of the grand average brain response (**A**) for the concussion (red) and control groups (black). Brain responses of concussed children are smaller over the consonant-vowel transition (**A**,**B**) and slower (**A**) than those of their non-concussed peers. Error bars represent ± 1 S.E.M.

Our next analysis considered the magnitude of the neural response over the CV transition. Children who sustained a concussion had smaller responses to speech than their uninjured peers (*t*(38) = 2.382, *p* = 0.022, Cohen’s *d* = 0.832; Fig. 2A/D). Because the response to the F_0_ dominates the time-domain FFR, we think this is a corollary of the diminished F_0_.

### Slower neural responses to speech following concussion

Our next analysis considered the *timing* of neural processing by asking how quickly the auditory system responds to several cues in the speech sound, which are represented by characteristic peaks in the FFR. The children who sustained a concussion had slower responses to some, but not all, stimulus features (group × peak interaction, *F*(5, 30) = 5.091, *p* = 0.002, η^2^ = 0.459; Fig. 2A). *Post-hoc* tests comparing individual peaks showed that children in the concussion group had responses nearly 0.4 ms slower than their uninjured peers for three of the six response peaks, reflecting the coding of the periodicity (note not all peaks were detectable in every child; Peak A, *t*(38) = 2.542, *p* = 0.015, Cohen’s *d* = 0.804; Peak D, *t*(36) = 2.258, *p* = 0.030, Cohen’s *d* = 746; Peak E, *t*(37) = 3.301, p = 0.002, Cohen’s *d* = 1.059; Fig. 2A). While a timing discrepancy of 0.4 ms is small, in the context of the subcortical auditory system it is clinically significant. These particular peaks reflect the coding of the F_0_^29^, suggesting that both the *magnitude* and the *timing* of F_0_-coding is disrupted.

However, the two groups had similar timing to the *onset* of the sound (Peak V, *t*(32) = 1.358, *p* = 0.183, Cohen’s *d* = 0.429), for the last peak reflecting the *transition* into a steady state vowel (Peak F, *t*(38) = 1.169, *p* = 0.250, Cohen’s *d* = 0.370), and in response to the *offset* of the sound (Peak O, *t*(36) = 0.876, *p* = 0.387, Cohen’s *d* = 0.284; Fig. 2A). Thus, it appears concussions impart a *selective* timing delay that only affects the neural coding of certain speech features. Specifically, it appears concussions target the coding of periodicity (F_0_) cues in speech while sparing transients (such as plosive onset bursts).

### Less accurate neural responses to speech following concussion

We correlated each individual’s FFR to the stimulus to achieve a “global” measure of the integrity of neural processing. Children in the concussion group, on average, had less accurate neural coding of the speech sound than their uninjured peers (*t*(38) = 2.660, *p* = 0.011, Cohen’s *d* = 0.841).

### Biomarker of concussion

The preceding analyses validated our predictions that (1) children with a concussion have poorer neural processing of sound than their peers, (2) this profile is grounded in neural processing of the F_0_, and (3) the integrity of this processing relates to the severity of the injury. Next, we asked if these physiological measures could be combined to classify children into concussion and control groups. If so, certain FFR properties could be used in aggregate as a biological marker to objectively and reliably identify a concussion.

We conducted a binary logistic regression, which asks how a series of measures combine to predict group membership. While the previous sections defined the specific neural functions that are disrupted in children with a concussion, here we were interested in evaluating how the FFR distinguishes between individuals with and without these injuries. We specifically wanted to see whether these objective biological factors could, in combination, identify the children in this study who had sustained a concussion. We used a two-step model that, on the first step, incorporated subject age, the background noise in the FFR (amplitude of non-stimulus-related neural activity), and the timing of the onset response to sound (wave V in response to a click). The second step incorporated the magnitude of the response to the F_0_, the size of the onset response (defined as the area between Peaks V and A), and the accuracy of encoding the speech sound (stimulus-response correlation). Our model correctly classified subjects into concussion or control groups (Log likelihood ratio = 23.028, Nagelkerke *R*^2^ = 0.741, *χ*^2^(6) = 32.423, *p* < 0.001; Table 2).

Finally, we evaluated the predictive utility of the FFR by conducting a receiver operating characteristic (ROC) analysis on scores from the logistic regression. We found that a cut-off of 0.596 on the regression score achieved a 90% sensitivity (true positive rate; 18 out of 20 mTBI subjects correctly classified) and a 95% specificity (true negative rate; 19 out of 20 control subjects correctly classified) was an excellent fit for the data (area under the curve = 0.945, *p* < 0.001, 95% confidence interval 0.875–1.000). These correspond to a 94.7% positive predictive value (PPV, probability that a positive is true) and a 90.4% negative predictive value (NPV, probably that a negative is true).

### Partial recovery of neural processing as concussion symptoms abate

Our final analysis focused on children who returned to the clinic for a second evaluation. If sound processing is disrupted by a concussion, then it follows that this processing should *improve* through the course of recovery. At the second test, all of the children reported a reduction in their symptom loads, suggesting that they were on the road to recovery (subgroup only; PCSS: Test 1, mean 37.1, SD 22.5; Test 2, mean 12.8, SD 15.2; *t*(10) = 4.342, *p* = 0.002). It is important to note, however, that only one of the children was clinically determined to be completely recovered from the concussion at this second visit and cleared to resume normal activities.

**Figure 3:**
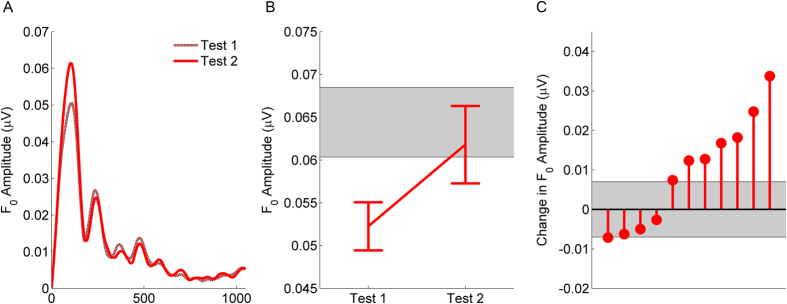
Longitudinal evidence shows that F_0_ processing improves as concussion symptoms abate. Between Test 1 and Test 2 (burgundy and red lines, respectively) the magnitude of responses to the F_0_ increases (**A**). The mean (±1 S.E.M.) of the concussion group at both test points. On average, they no longer differ from the control group with respect to F_0_ processing (mean ± 1 S.E.M. showed as gray shaded region) (**B**). Although 5 of the subjects are within this range, 6 show increases beyond that range. Changes in F_0_ amplitude for individual subjects from the concussion group are shown (**C**). The shaded gray area shows the range F_0_ amplitude that would indicate chance level of change based on normative data.

In line with this reduction in symptom load, we found that F_0_ responses were ~30% larger at the second test, whereas responses to the harmonics remained the same (test × frequency interaction, *F*(1, 10) = 6.287, *p* = 0.031, η^2^ = 0.386; F_0_: *t*(10) = 2.397, *p* = 0.037; harmonics: *t*(10) = 1.453, *p* = 0.177). As illustrated in Fig. 3B, the re-test group’s F_0_ amplitude matched the range of the control group.

We also computed the minimal detectable change in F_0_ amplitude based on published norms^25^; this provides a cutoff for a change in F_0_ amplitude that would be more than expected by chance^31^. We found that 6 of the 11 children in this group improved in F_0_ amplitude beyond chance (change of 0.006 μV). As shown in Fig. 3C, of the five that did not significantly increase in F_0_ amplitude, none declined significantly. While this is a small subsample, this longitudinal evidence for F_0_ recovery provides a converging proof-of-concept that reinforces our cross-sectional findings.

## Discussion

We tested the hypothesis that concussions disrupt the neural processing of speech. Children with a concussion exhibit a signature neural profile that distinguishes them from their non-concussed peers. This profile is manifest in the neural coding of a specific ingredient of sound: following a concussion, neural responses process the F_0_ of speech less robustly and are smaller, slower, and less accurate. The symptom load of an injury relates to this neural profile—concussed children with the highest symptom loads have the weakest responses to speech. We also show that the FFR reliably identifies which children sustained a concussion, suggesting its clinical potential as a biological marker of an injury. These findings are reinforced by partial recovery of F_0_-coding as concussion symptoms abate. Note, however, that aspects of this profile are *selective* for certain aspects of neural processing—for example, the groups had similar timing in response to the sound’s onset and offset, and processed the harmonics of speech similarly. Together, these results support the idea that concussions disrupt the neural processing of F_0_-bearing information, which is critical for identifying, grouping, and tracking sounds—a key element of listening in complex environments. Thus, this F_0_-based neural signature ties into previous behavioral studies showing declines in auditory processing following traumatic brain injuries^8^.

### From neural processing to everyday communication

It is interesting to note several similarities between this neural signature of concussions and the neural roots of speech perception in noise. The strength of coding the F_0_ in speech underlies successful speech understanding in noise across the lifespan^10^,^11^. The F_0_ is a chief acoustic cue that conveys the pitch of a sound, a factor in auditory object formation that allows a listener to hone in on a voice against a din—for example, distinguishing and tracking male *vs.* female voices. Also noteworthy is that Peaks D and E, which we find are slower in children with a concussion, reflect this periodicity coding (i.e. coding of F_0_). Because mild traumatic brain injury (mTBI) is associated with difficulty understanding speech in noise^8^,^9^, our finding of poor F_0_ representation (both cross-sectionally and its recovery longitudinally) is consistent with the view that mTBI compromises auditory processing and the ability to make sense of sound. These findings hint at the biological mechanisms underlying this observation. These findings are also consistent with the broader idea that concussions disrupt sensory processing (including in the auditory, visual, and somatosensory systems) concomitant to cognitive deficits^32^.

### Concussion pathophysiology: A view from the auditory system

Concussion pathophysiology is heterogeneous and not yet entirely understood. A number of mechanisms may contribute to the auditory-neurophysiological profile we have identified, and we highlight several as hypotheses for future work. FFR properties are shaped by bottom-up, local, and top-down factors^13^ and its generators have been studied extensively in humans and animal models. Our results therefore provide clues to the biological factors that may be disrupted following a concussion.

In the auditory periphery, acute noise trauma degenerates primary afferents and causes synaptic swelling and bursting, which is thought to interfere with everyday listening^33^; sudden head impact may cause axonal shearing that damages cochlear afferents and/or the auditory nerve, degrading responses to the amplitude modulations in speech, such as the F_0_^34^. Demyelination could also delay neural transmission and diminish population response magnitude, consistent with our findings.

mTBI has also been associated with diminished levels of glutamate^35^, an excitatory neurotransmitter. The fine temporal resolution in the auditory system relies on a balance of inhibitory and excitatory neurotransmission^36^, and it has been hypothesized that an imbalance creates variability in first-spike latency in auditory midbrain^37^ (which could contribute to the abnormalities we observed in the onset response, peak A) and to sluggish peak timing in response to dynamic speech features (which could contribute to the abnormalities we observed in response to the consonant-vowel transition, peaks D and E^38^).

Finally, it is important to recognize that FFR properties are shaped by experiences and cognitive activity^13^, and corticofugal fibers from temporal and prefrontal cortices project directly to auditory midbrain to modify response properties^24^. Temporal and frontal cortices are thought to be the areas of neocortex most susceptible to injury in concussions^32^, and mTBI may cause abnormal gain mechanisms for subcortical neural coding. Given links between F_0_ strength and attention skills^39^, this may contribute to the poor pitch processing we observe and the broader phenotype of poor auditory processing in individuals with mTBI.

Of course, these remain open questions. Should future studies confirm that the FFR is a valid marker for mTBI, this would open the door to mechanistic studies. For example, by combining FFR markers with a thorough behavioral battery, blood markers, and neuroimaging, a more sophisticated mechanistic picture of mTBI could be drawn.

### Clinical potential

In the clinic, concussions present a distinct set of challenges. A concussion is a clinical diagnosis that requires a physician to evaluate a constellation of potential symptoms across multiple organ systems^32^. It is nearly impossible to predict how long a patient will suffer from a concussion—although many cases resolve within a few days, others last from months to years^40^. Athletes with concussions cannot safely return to play until these symptoms have resolved, so the uncertainty about the length of recovery often adds to patients’ stress, and may contribute to development of anxiety and depression symptoms following a concussion. Here we show that the FFR identifies a concussion with a 94.7% PPV and 90.4% NPV. For comparison, the ImPACT—a widely-used behavioral test battery—has an 89.4% PPV and 81.9% NPV^41^. Similarly, the Standardized Assessment of Concussions has a 91.2% PPV and an 83.1 NPV^42^. Although further study is needed, including validation in a novel cohort to determine this model’s generality, this suggests the FFR may reliably indicate a concussion and clear a non-concussion. Additionally, the FFR tracks recovery. Together, this suggests the FFR’s potential as a clinical adjunct in concussion management.

### Limitations

Some limitations should be acknowledged. First is the tertiary-care clinic setting that may have biased our sample to the concussion cases with higher symptom loads and more prolonged recoveries; an important next step is to replicate these findings in a cohort with less severe concussions. Similarly, our longitudinal analyses consisted of children whose concussions lasted the longest, meaning these are even more strongly biased to severe concussion cases. We also acknowledge the modest sample size; an important next step is to replicate these findings in a larger and more diverse population.

These findings motivate future experiments to understand how concussions jeopardize the ability to make sense of sound, and how auditory-neurophysiological testing may provide a clinical adjunct for diagnosis and management. For example, to determine causality, a prospective study that follows athletes over the course of a season is needed. Ideally it would measure an athlete’s baseline FFR at the beginning of the season, and then again following a concussion during the season, and compare outcomes to age-matched athletes from the same team who did not get concussed during the season. Our longitudinal finding of a partial recovery provides some causal support for our hypothesis, but our interpretation is still constrained by the lack of a baseline, and the lack of a test on all subjects once they are fully recovered and cleared to resume all activities.

### Future directions

The FFR is mobile, has high test-retest stability, and may be administered many times to the same individual. Thus, it could determine when a player has returned to his/her “personal baseline” and therefore indicate when it is safe to return to activity. Importantly, unlike other clinical tools for concussion evaluation (self-reported symptoms, computerized neurocognitive tests, balance tests, and visual skills tests), the FFR is objective and not dependent on the subject’s effort or truthfulness in reporting (FFR results cannot be controlled or manipulated by the subject). Although our emphasis here was sports-related concussion, our approach has potential as a clinical adjunct in a variety of non-penetrating head injuries, including blast-induced injury and the broader spectrum of mTBI, in addition to their rehabilitation^43^,^44^. While further study is necessary, this approach has potential as a scalable, practical assessment for mTBI.

One aspect of mTBI this study did not address is its behavioral consequences. Disruptions to speech perception, attention, memory, and processing speed have all been reported following a concussion^2^,^8^,^32^; tracking these is an important aspect of their management. Similarly, pre-existing differences in cognitive or listening skills could account for the group differences we identify. Future investigation into what relationship exists between the behavioral and FFR sequelae of mTBI is warranted, especially work that tries to understand any potential mechanistic relationship. Given evidence of bidirectional input between sensory and cognitive systems^13^, the FFR signature we outline may itself be a consequence of disruptions to cognitive functions.

Further longitudinal study could address the questions of predicting susceptibility to a concussion and/or the time course of recovery. Finally, we note an important line of research in sports-related concussions that investigates strategies to prevent injuries or reduce their severity, through the development of new athletic techniques, regulations, equipment, and playing fields. The FFR may be useful in efficacy studies of concussion prevention.

Together, this report defines a new neural signature of concussions that, in a majority of cases, pinpoints a bottleneck in sound processing. Early evidence shows this signature partially recovers as concussion symptoms abate. From a theoretical standpoint, these findings illustrate how auditory processes are susceptible to neurological insults and hint at the pathophysiology underlying difficulties in everyday listening. From a clinical standpoint, these findings define a new measure that may assist in diagnosis and management of concussions.

## Methods

### Ethical Statement

All study procedures were approved by the Institutional Review Boards of Ann & Robert H. Lurie Children’s Hospital of Chicago and Northwestern University, concordant to the Declaration of Helsinki. Following a description of the study procedures, parents/guardians provided written informed consent for their children to participate in this study and the children aged 12 and older provided written assent.

### Experimental Design & Subjects

Two groups of children participated in this study. Inclusionary criteria included normal hearing, no neurologic disease, and no history of severe TBI. Descriptive statistics for both groups are provided in Table 1. Because we predicted that concussions would disrupt F_0_ processing, we wanted a sufficient number of subjects to detect group differences in the F_0_^12^—hence forty total subjects.

The concussion group (*N* = 20, 6 males, mean age = 13.39 yr, SD = 1.79 yr) was recruited from the Institute of Sports Medicine at Ann & Robert H. Lurie Children’s Hospital of Chicago, a specialty clinic. Children in this group met clinical diagnostic criteria for a concussion^45^ and participated in the experiment following their medical evaluation by a sports medicine physician (CRL) with expertise in concussion diagnosis and management. On average, they were evaluated 27 days after their injury (mean = 26.7 days, SD, 15.3 days, range: 6–56 days). Injuries were attributed to basketball (*N* = 1), cheerleading (*N* = 2), football (*N* = 3), hockey (*N* = 2), soccer (*N* = 1), softball (*N* = 2), volleyball (*N* = 1), and other recreational activities (*N* = 8). Thirteen of the children reported a history of a previous concussion. Six of the children had a computerized tomography (CT) scan of the head and two had magnetic resonance imaging scans of the head; all were normal, except one had a preexisting cyst and one child had a slight odontoid asymmetry. Because the concussion group was recruited and tested at a specialty clinic the data collection could not be blinded to subject group.

A subset of the subjects from the concussion group returned to the clinic for follow-up and were retested (N = 11, 3 males, mean age = 13.25 yr, SD 1.93 yr; average test-retest interval: 34.9 days, SD 15.7 days, range 11–56 days). Although only one of the subjects was fully *recovered* at this visit, they were seen by the clinical team as a part of concussion monitoring. Two of them reported that their symptoms had abated since the first evaluation, and at the second evaluation one was cleared to resume normal activities.

The control group (*N* = 20, 6 males, mean age = 13.64 yr, SD 1.87 yr) was recruited from the community through in-school flyers and word of mouth; none reported a history of brain injury. The groups were matched with respect to age (*t*(38) = 0.092, *p* = 0.927, Cohen’s *d* = 0.029) and had the same distribution of males and females.

All the subjects passed a hearing screening involving distortion product otoacoustic emission screening, suggesting normal outer hair cell function in the cochlea (>6 dB signal-to-noise ratio from 0.4–5 kHz). All subjects had normal auditory brainstem responses (ABR) to a 100 μs click presented at 80.4 dB SPL to the right ear, and the two groups had similar ABR onset response timing (Wave V: *t*(38) = 0.261, *p* = 0.795, Cohen’s *d* = 0.083). The comparable click-evoked response timing suggests that concussions do not compromise signal transduction through the peripheral auditory pathway; this observation is consistent with previous research^9^.

### Neurophysiology

Frequency-following responses (FFRs^20^) were elicited by a 40 ms sound /d/ synthesized in a Klatt-based synthesizer (SenSyn, Sensimetrics Corporation, Malden, MA). The stimulus begins with a plosive burst during the first 10 ms, with a 5 ms voice-onset time. During the voiced period of the stimulus, the fundamental frequency (F_0_) rises linearly from 103 → 125 Hz while the formants shift linearly as follows: F_1_ 220 → 720 Hz, F_2_ 1700 → 1240 Hz, and F_3_ 2580 → 2500 Hz. The last two formants are steady throughout the stimulus (F_4_ 3600 Hz, F_5_ 4500 Hz). Although the stimulus is brief and there is no vowel, it is perceived as the consonant-vowel syllable [da]. Normative data are available for responses to this stimulus in normal-hearing individuals from birth to age 72 yr^25^.

Stimuli were delivered and responses were collected through a Bio-logic Navigator Pro System (Natus Medical Inc., Mundelein, IL). FFRs were measured in a vertical montage with three Ag-AgCl electrodes (Cz active, Fpz ground, right earlobe reference). Stimuli were delivered to the right ear in alternating polarities at 80.4 dB sound pressure level at 10.9 Hz through an electromagnetically-shielded insert earphone (Etymōtic Research, Elk Grove Village, IL). Responses were filtered online from 100–2000 Hz (second-order Butterworth) and sampled at 12 kHz. Online artifact rejection was employed at ± 23 μV, and two blocks of 3000 artifact-free stimulus presentations were averaged with a 75 ms recording epoch (including a 15.8 ms non-stimulus period, which served as a control measure of background noise).

### Concussion Symptom Severity

Children in the concussed group completed the Postconcussion Symptom Scale (PCSS^46^,^47^) to report their symptom load. For each of the 19 symptoms, encompassing neurocognitive, emotional, and somatic aspects of concussion symptomology, subjects indicated on a Likert scale of 0–6 the intensity of each symptom. The PCSS total score is the sum of the scores for each symptom, and represents the subject’s symptom load. Higher scores reflect greater symptom loads. Total PCSS scores at test one ranged from 0 to 71 (mean 31.5, SD 21.9) and, in the eleven children who returned for a follow-up test, from 0 to 52 at test two (mean 12.8, SD 15.2). Headache was the most frequently reported symptom (test one: 17/20 concussion patients; follow-up test: 7/11 patients). Also reported were difficulty concentrating (test one: 16/20 concussion patients; follow-up test: 7/11 patients), drowsiness (test one: 14/20 concussion patients), and photosensitivity (test one: 14/20). No patients reported nausea or vomiting.

### Data Analyses

Neurophysiological responses were analyzed with respect to *magnitude, timing, accuracy,* and *F*_*0*_*/pitch processing*^20^,^25^. The fundamental frequency (F_0_) amplitude was defined as the spectral amplitude between 75 to 175 Hz, which corresponds to the F_0_ of the stimulus; this was compared to harmonic coding (175–750 Hz). To determine spectral amplitudes, the response was converted to the frequency domain (from 19.5 to 44.2 ms; fast Fourier transformation with a 2 ms Hanning window). As a complementary analysis to determine the strength of pitch coding, an autocorrelation was run from 19.5 to 44.5 ms (sliding window, 20 ms bins, 1 ms of overlap) and the mean correlation at a lag corresponding to the average period of the stimulus was determined^30^. To determine response magnitude over the consonant-vowel transition, the root-mean-squared amplitude of the response was computed from 19.5 to 44.2 ms (corresponding to the voiced period of the stimulus). To determine timing, the latencies of several stereotyped response peaks were identified. Peaks were identified on the final average in consultation with a normative template and two subaverages. To determine accuracy, the stimulus was filtered to match the response (100–2000 Hz, second-order Butterworth) and each child’s response was cross-correlated to the filtered stimulus (from 19.5 to 44.2 ms; the maximum correlation at an appropriate lag was obtained and Fisher-transformed to *z* scores for statistical purposes). All statistics reported reflect two-tailed tests.

## Additional Information

**How to cite this article**: Kraus, N. *et al*. Auditory biological marker of concussion in children. *Sci. Rep.* **6**, 39009; doi: 10.1038/srep39009 (2016).

**Publisher's note:** Springer Nature remains neutral with regard to jurisdictional claims in published maps and institutional affiliations.

## Figures and Tables

**Table 1:** Descriptive statistics for the concussion and control groups.

		Control (*N* = 20)	Concussion (*N* = 20)	Concussion – Retest (*N* = 11)
Age (yr)		13.64 (1.87)	13.69 (1.79)	13.25 (1.29)
Male:Female		6:14	6:14	3:8
Click V Latency (ms)		5.64 (0.22)	5.66 (0.22)	5.66 (0.30)
Magnitude over CV Transition (μV)		0.09 (0.02)	0.07 (0.03)*	0.07 (0.04)
Timing (ms)	V	6.52 (0.25)	6.62 (0.24)	6.82 (0.41)
	A	7.42 (0.27)	7.65 (0.31)*	7.82 (0.41)
	D	22.29 (0.30)	22.63 (0.59)*	22.80 (0.92)
	E	30.80 (0.40)	31.23 (0.44)**	31.09 (0.30)
	F	39.37 (0.35)	39.52 (0.44)	39.55 (0.52)
	O	48.24 (0.37)	48.13 (0.38)	48.18 (0.41)
Stimulus-response correlation (Pearson’s *r*)		0.15 (0.10)	0.08 (0.04)*	0.08 (0.05)
Spectral magnitude (μV)	F_0_	0.068 (0.017)	0.048 (0.019)***	0.062 (0.015)^†^
	Harmonics	0.019 (0.005)	0.017 (0.007)	0.018 (0.004)
Pitch coding (Pearson’s *r*)		0.30 (0.08)	0.24 (0.07)**	0.24 (0.09)

Means are reported with standard deviations.

Concussion vs. Control group: *p < 0.05, **p < 0.01, ***p = 0.001; Concussion Subgroup Test 1 vs. Test 2 ^†^*p* < 0.05.

**Table 2:** A binary logistic regression that incorporates multiple aspects of auditory-neurophysiological processing reliably classifies 90% of children into concussion or control groups.

		B	S.E.	Wald χ^2^
Step 1	Age	−0.24	0.33	0.52
	Prestimulus amplitude	−11.26	63.67	0.03
	Wave V ABR latency	2.545	2.99	0.72
Step 2	Onset magnitude	40.14	17.54	5.33*
	F_0_ magnitude	−161.82	59.74	7.34*
	Stimulus-response correlation	−20.83	8.63	5.82**

*p < 0.05, **p = 0.01.
